# Prevalence, Patterns, Severity, and Sociodemographic Correlates of Childhood Sexual Abuse Among Adults with Hypertension

**DOI:** 10.3390/healthcare14111503

**Published:** 2026-05-28

**Authors:** Turki S. Alqurashi, Abrar I. Aljohani

**Affiliations:** 1 Department of Social Work, Al-Lith University College, Umm Al-Qura University, Makkah 28429, Saudi Arabia; 2 Department of Clinical Laboratory Sciences, College of Applied Medical Sciences, Taif University, Taif 21944, Saudi Arabia; abrar.g@tu.edu.sa

**Keywords:** childhood sexual abuse, CSA, hypertension, children’s health, well-being

## Abstract

**Background:** The prior literature associates childhood sexual abuse (CSA), a profound early-life stressor, with long-term cardiometabolic and psychological effects. However, data on the distribution of CSA severity and patterns among different sociodemographic groups are limited. This study aimed to describe CSA prevalence, patterns, and severity among adults with hypertension and to examine its association with sociodemographic characteristics. **Methods**: This cross-sectional study evaluated a sample of 353 adults using various self-reported items. The analysis used individual responses and a binary variable indicating CSA exposure. A composite CSA severity score was determined by the number of reported abuse patterns and categorized into ordinal levels. **Results**: Of the participants, 42.2% reported exposure to CSA. The most frequently reported pattern was sexual contact or harassment (26.1%), followed by other sexual abuse acts (25.2%), forced sexual acts or exposure (18.7%), and coerced sexual activity (17.3%). Sexual assault or rape was reported by 20.7% of respondents. In terms of severity, 57.8% of respondents reported no CSA exposure, whereas 10.8% reported experiencing the highest severity. All examined sociodemographic factors were significantly associated with CSA exposure (*p* < 0.001). **Conclusions**: High CSA exposure and greater severity patterns were prevalent in this population, demonstrating notable sociodemographic gradients. These findings support the need for trauma-informed therapeutic awareness of CSA exposure among adults with hypertension. Nevertheless, the role of CSA as a risk factor for hypertension cannot be inferred from this study, as it only included subjects with preexisting hypertension. Longitudinal research is necessary to identify causal pathways.

## 1. Introduction

Child sexual abuse (CSA) is defined as “the involvement of a child (person less than 18 years old) in sexual activity that the child does not fully comprehend, cannot give consent or violates the laws or social taboo of society” [1]. While frequently concealed and underreported, international data suggest that CSA represents a widespread violation of children’s rights. Historically, some societies regarded sexual contact between adults and children as a cultural or educational practice; however, contemporary perspectives increasingly recognize CSA as a severe form of violence and a public health priority [2,3]. Recent reviews report variable but consistently high prevalence estimates worldwide. Nordic countries report rates of approximately 3–23% for boys and 11–36% for girls, while in Japan, rates range from 1% to 64% for girls and 0% to 44% for boys. In India, rates range from 4% to 41% for girls and 4% to 57% for boys [4,5,6], and in Australia, 37.3% of women, 18.8% of males, and 52% of gender-diverse individuals have reported a history of CSA [7,8]. Recent research in Saudi Arabia suggests a CSA prevalence of 10–15% in school-based samples of teenagers and of up to 30% in certain young-adult samples [9,10].

CSA has extensive adverse effects on families and children. It has a detrimental impact on children’s mental health, leading to conditions such as major depressive disorder, post-traumatic stress disorder, borderline personality disorder, anxiety, psychosis, eating disorders, and conversion disorder [11,12,13]. CSA is also associated with psychological issues such as substance misuse, self-harm, suicide attempts, inappropriate sexualized conduct, and high-risk sexual behaviors [11,12,13]. Furthermore, the consequences of CSA extend to family members, who may experience emotional stress, disturbed relationships, and vicarious traumatization. Ultimately, these impacts may profoundly affect personal and professional functioning, leading to social isolation and increased job absenteeism [14].

In addition to its significant psychological effects, CSA is linked to several deleterious physical health outcomes in adulthood, including cardiometabolic disorders [15,16]. Childhood maltreatment—and CSA in particular—has been associated with an elevated risk of obesity, impaired glucose metabolism, and type 2 diabetes. This elevated risk may be attributed to chronic stress pathway activation and various behavioral mediators, including smoking, physical inactivity, and poor diet [15,17,18].

Large-scale cohort and population-based studies have established that individuals subjected to childhood maltreatment, including sexual abuse, exhibit increased odds of elevated blood pressure and adult-onset hypertension, even after controlling for conventional risk factors [19,20]. Research in North American and European populations has revealed dose–response correlations between the severity or frequency of childhood maltreatment types and subsequent hypertension or unfavorable cardiometabolic profiles [19,20,21]. However, such studies predominantly focus on Western populations and frequently utilize community samples, and research specifically addressing the patterns and severity of CSA in adults with hypertension is scarce, as are investigations of the relationship between CSA and sociodemographic characteristics within these clinical cohorts [16]. There is a particularly evident research gap in the Middle Eastern and Gulf regions, such as Saudi Arabia, where both CSA and hypertension are increasingly problematic yet remain insufficiently studied in conjunction [22].

The present study therefore seeks to address a crucial knowledge gap at the crossroads of CSA and chronic non-communicable disease by characterizing the prevalence, patterns, and severity of CSA among adults with hypertension in Saudi Arabia, as well as exploring its association with sociodemographic factors.

## 2. Materials and Methods

### 2.1. Study Population and Eligibility Framework

This cross-sectional study recruited adults (≥18 years) in Saudi Arabia who had been diagnosed with hypertension. Eligibility criteria included current use of antihypertensive medication or a doctor’s confirmation of Stage 1 or Stage 2 hypertension. To ensure the clinical specificity of the research population, individuals with prehypertension or pregnancy-associated hypertension were excluded. To improve inclusivity across adult age groups, no upper age limit was imposed. Based on a 95% confidence level, a 5% margin of error, and a conservative response distribution of 50%, the Raosoft calculator was used to determine a minimum required sample size of 377 participants. During the data collection period, 353 eligible respondents completed the survey. Low enrollment was anticipated due to the sensitive nature of the study’s combined emphasis on chronic disease and sexual assault. Nevertheless, the final sample size was sufficient to substantiate the exploratory and multivariable association analyses.

### 2.2. Recruitment Strategy and Data Collection Procedures

Data were collected using structured online questionnaires administered via Google Forms between May 2025 and April 2026. We used a community-oriented digital dissemination method to recruit individuals with hypertension in Saudi Arabia. To increase targeted reach, survey links were sent via institutional communication channels, chronic illness support groups, hypertension-related community networks, and social media platforms.

Clinic-based recruitment via cardiology outpatient services, primary health care facilities, and accredited cardiology charities was considered, but was ultimately deemed impracticable given the study’s inclusion of very sensitive inquiries about CSA and exposure to violence. Digital community recruiting was thus selected as the most appropriate and ethical approach. Eligibility screening was implemented at the start of the survey. Participants were required to verify their age (≥18 years) and whether they had hypertension (Stage 1 or Stage 2 diagnosis or usage of antihypertensive medication). Exclusion criteria were systematically applied via screening questions, allowing only qualified respondents to proceed to the full questionnaire. The survey platform employed mandatory-response settings to reduce item-level missingness; only fully completed surveys were therefore included in the analysis.

### 2.3. Measurement Approach and Instrument Adaptation

Exposure to CSA was evaluated using a culturally adapted version of the sexual abuse subscale of the Childhood Trauma Questionnaire–Short Form (CTQ-SF) [23]. Given that instruments developed for Western contexts may not be immediately applicable to conservative sociocultural contexts, we applied linguistic and cultural modifications to several questions in order to enhance the interpretability and validity of the standardized abuse assessment instruments in a Saudi population. Consistent with established cross-cultural validation protocols, the adaptation process included forward–backward translation by bilingual experts, a multidisciplinary panel review involving social science and public health specialists, and cognitive assessment with 15 Saudi individuals. Minor wording refinements were made to enhance cultural acceptability and preserve conceptual equivalence. The assessment approach aimed to capture various characteristics of CSA experiences, such as intensity, patterns, and prevalence. Despite the potential for direct cross-national comparability of data collected using multidimensional instruments that have been internationally standardized, culturally adapted approaches may enhance the disclosure sensitivity, participant acceptability, and contextual appropriateness in conservative sociocultural contexts, such as Saudi Arabia [24].

To facilitate disclosure and alleviate respondent burden regarding the sensitive questions, a binary response format (yes/no) was employed to administer the items. Pilot testing confirmed strong face validity, and construct adequacy was supported by expert consensus. The adapted subscale demonstrated a high level of internal consistency (Cronbach’s α = 0.87). The questionnaire was divided into two sections: sociodemographic variables (age, gender, education, occupational status, and marital status) and a six-item CSA questionnaire.

### 2.4. Ethics Approval and Participant Safeguards

This study was conducted as part of a more extensive, ethically approved research program investigating the correlations between non-communicable diseases and family violence (including child maltreatment and intimate partner violence). Prior to data collection, ethical approval was granted by the Biomedical Research Ethics Committee from Umm Al-Qura University (approval no. HAPO-02-K-012-2025-03-2622).

Due to the sensitive nature of the survey questions, participation was voluntary, and the questionnaire was accompanied by an electronic informed consent statement outlining the study’s aims, methods, content sensitivity, and withdrawal options. Participants were advised that they may refuse to answer any question or quit the survey at any time without penalty. For individuals potentially experiencing emotional discomfort, the survey included information on accessible psychological support options. No personally identifying information was collected, thereby reducing privacy concerns and promoting personal disclosure. The online, self-administered nature of the survey allowed participants to choose the time and place of their participation.

Data were stored in encrypted, access-controlled files that were restricted to the research team. All procedures were implemented in accordance with the institution’s ethical standards for the preservation of human subjects, which encompassed standards regarding participant privacy, confidentiality, and safety. Established ethical standards of beneficence and respect for individuals were implemented across all procedures, and the study adhered to the STROBE guidelines for cross-sectional investigations.

### 2.5. Statistical Analysis

The data were analyzed using IBM SPSS Statistics (version 29). Categorical data were summarized using frequencies and percentages. CSA exposure variables were coded as either “yes” or “no” for each abuse pattern. CSA was evaluated using various self-reported items and summarized using both individual responses and a composite binary variable indicating any CSA exposure. A CSA severity score was created by summing the number of positive abuse pattern responses reported by each participant, yielding a composite severity index divided into ordinal levels (0–4). Given that comprehensive information on frequency, length, level of coercion, or contextual severity was not provided to warrant differential weighting, each abuse category was weighted equally. Participants were then grouped into ordinal severity categories for descriptive interpretation of the cumulative burden of exposure. This pragmatic method was considered an experimental, not validated, clinical severity scale.

The chi-square test of independence (or Fisher’s exact test for predicted cell counts <5) was used to assess the association between CSA exposure (yes/no) and sociodemographic factors. Given that the study outcome (history of sexual abuse: yes/no) was binary, multivariable logistic regression was performed to identify characteristics independently related to the outcome. Variables were chosen based on conceptual significance and univariable results. The final model included sex, marital status, and employment. Age was considered throughout the model construction but was deleted from the final model because its inclusion resulted in a worse model fit, and it did not substantially enhance model performance. The omnibus test, Hosmer–Lemeshow goodness-of-fit test, Nagelkerke R^2^, and classification accuracy were used to evaluate model adequacy. Adjusted odds ratios (aORs) with 95% confidence intervals (CIs) are presented. All tests were two-sided, and a *p*-value < 0.05 indicated statistical significance.

## 3. Results

### 3.1. Participant Characteristics

This study included 353 participants with hypertension, with no missing data. Most participants were women (74.8%), and most were married (43.9%). Approximately 50% of the participants were over 50 years old, and most held a bachelor’s degree. Table 1 summarizes the participants’ various sociodemographic characteristics.

### 3.2. Prevalence and Patterns of Childhood Sexual Abuse

Overall, 42.2% of the participants reported experiencing at least one form of CSA. The most frequently reported pattern was sexual touching or harassment (26.9%), followed by force to engage in sexual activity (17.3%) and forced sexual acts or exposure (18.7%). There were also numerous disclosures of other sexual abuse (25.2%). Additionally, 20.7% stated that they had been sexually abused or raped before receiving their hypertension diagnosis. The CSA severity assessments revealed that 57.8% of participants had no CSA exposure, with the remainder distributed across varying degrees of severity, including 10.8% at the highest level (Table 2).

### 3.3. Associations Between Sociodemographic and Childhood Sexual Abuse

Table 3 illustrates significant correlations between CSA and all analyzed sociodemographic variables (all *p* < 0.001). A higher proportion of females reported CSA than males—females comprised 85.2% of the CSA-exposed group and 67.2% of the non-exposed group (χ^2^ = 14.92, *p* < 0.001).

The age distributions differed across categories, with a higher prevalence of CSA exposure among individuals aged 50 years or older (57.7%). CSA exposure was significantly higher among married participants (70.5%) compared with single (14.8%) and divorced (14.8%) individuals (χ^2^ = 78.86, *p* < 0.001).

Similarly, educational level was associated with CSA status, with a greater proportion of CSA-exposed participants (70.5%) holding a bachelor’s degree, whereas no participants with higher degrees were classified as CSA-exposed (*p* < 0.001). Additionally, a significant disparity was observed in occupational status; the prevalence of CSA was highest among the retired (44.0%) and the unemployed (27.5%), with only 14.8% of students and 14.8% of the employed reporting exposure (*p* < 0.001).

### 3.4. Multivariable Logistic Regression Analysis

The multivariable logistic regression model was statistically significant (Omnibus χ^2^ = 105.67, *p* < 0.001), showing that the prediction was better than that of the null model. The model had an adequate fit (Hosmer–Lemeshow χ^2^ = 5.56, *p* = 0.235), suitable explanatory power (Nagelkerke R^2^ = 0.348), and overall classification accuracy of 73.4%.

Males were significantly more likely than females to disclose a history of sexual abuse (OR = 4.68, 95% CI: 2.45–8.95; *p* < 0.001), and married individuals had significantly higher odds compared with unmarried individuals (OR = 11.96, 95% CI: 6.56–21.81; *p* < 0.001). After adjustment, occupation was not significantly correlated with the outcome (OR = 1.64, 95% CI: 0.83–3.26; *p* = 0.157) (Table 4).

## 4. Discussion

This study investigated CSA prevalence, patterns, and severity, as well as its associations with sociodemographic variables in a Saudi adult population diagnosed with hypertension. Of the participants, 42.2% reported having been sexually abused. They reported various forms of abuse, with sexual contact/harassment being the most frequent. A substantial proportion of reported forms of abuse fell into the more severe categories. We also observed statistically significant correlations between CSA exposure and sex, age group, marital status, education level, and occupation. In the multivariable analysis, associations between exposure to CSA and selected sociodemographic characteristics remained significant after adjusting for measured confounders. Sex and marital status were independently correlated with reported CSA, suggesting the possible role of social and contextual variables in exposure, disclosure patterns, or both. No independent correlation was found after adjustments between CSA and occupation. The results should be considered associative and not causative due to the cross-sectional methodology.

The CSA prevalence in our study population was within the upper range documented across other nations. Meta-analyses and extensive population surveys indicate that the prevalence of CSA ranges from 10% to 35%, depending on the measurement techniques employed, criteria applied, and cultural context [25,26]. Research employing behavior-specific questionnaires and anonymous self-administered surveys tends to yield elevated prevalence estimates, as these techniques mitigate underreporting [27]. The use of multiple behavior-based CSA items in this study may explain its higher reported CSA prevalence compared with studies using single-item screening questions.

Our findings indicate that females are significantly more likely to disclose CSA exposure, which is consistent with previous results [25,28]. Numerous global and regional studies have found that females report a higher burden of sexual abuse, despite male CSA being recognized as underreported due to shame, gender norms, and disclosure barriers [29]. The gender difference observed in our study aligns with established epidemiological trends while also highlighting the possibility that male CSA remains underestimated [30]. Consequently, the observed gender disparities in CSA prevalence may partially reflect variations in disclosure and unreported CSA exposure, particularly among men, rather than exposure differences. This potential under-detection has significant implications for clinical practice, as an increasing body of evidence indicates that sexual abuse during infancy and other early-life factors are associated with an increased long-term risk of cardiometabolic disease, including hypertension [19,31]. The mechanisms proposed in the literature as potential pathways linking early-life adversity to adult cardiometabolic consequences include chronic stress activation, dysregulation of the hypothalamic–pituitary–adrenal axis, autonomic imbalance, inflammation, and detrimental health behaviors. CSA may be a significant early-life adversity related to long-term health; however, its significance in the development of hypertension cannot be evaluated from this cross-sectional investigation [32].

Our analysis also found age-related discrepancies in reported CSA exposure, with older age groups (≥50 years) reporting higher rates. This pattern has also been documented by retrospective research and may be attributable to cohort effects, variations in memory, survivability, or generational differences in the degree of readiness to discuss abuse experiences. It may also reflect a greater potential for exposure over time or changes in societal awareness and reporting norms [29,33]. The significant correlations found between CSA and marital, educational, and occupational status are consistent with the literature linking early-life trauma to adverse long-term social and economic outcomes [34]. Longitudinal and population-based studies have documented associations between childhood abuse and relationship instability, lower educational attainment, and employment difficulties [35]. These associations may reflect the mental health effects of trauma and toxic stress, which impact cognitive, emotional, and social functioning throughout the lifespan [36]. The same stress-mediated biological and behavioral pathways have also been linked to an increased risk of cardiometabolic disease (including hypertension) through chronic neuroendocrine activation, inflammation, and negative coping behaviors [37]. However, given the cross-sectional nature of the present study, these associations should only be interpreted as correlational rather than causal.

The results can also be interpreted considering the WHO trauma-informed frameworks for violence and abuse surveillance. While acknowledging the significant long-term physical and psychological repercussions linked to childhood trauma exposure, these frameworks, along with the larger body of literature on adverse childhood experiences, emphasize safe, confidential, and culturally sensitive approaches to violence-related data collection. To ensure the safety of participants and the sensitivity of disclosure, this study employed culturally adapted terminology, voluntary informed consent, and anonymous participation. Additionally, the structured evaluation of the prevalence, patterns, and intensity of CSA contributes to broader public health surveillance efforts by supplying preliminary data from an underrepresented sample within the Saudi context [18,38].

Furthermore, the severity distribution observed in this study is notable. Although most respondents reported no CSA exposure, a significant subgroup was classified in the higher severity category, indicating that they had been exposed to multiple abuse patterns. Research has consistently demonstrated a dose–response relationship between the severity and accumulation of childhood maltreatment exposures and subsequent adverse physical and mental health outcomes [39,40]. The analytical and prognostic value of severity-based exposure measures in risk modeling is supported by research demonstrating an association between higher adversity scores and progressively increased risk of cardiometabolic diseases, including hypertension [31,37]. However, the severity scale for CSA used in this study should be interpreted with caution. It was based on a basic count of patterns of abuse recorded; thus, all experiences were treated equally, although various types of abuse may differ significantly in their severity and effect. In contrast to validated instruments such as CTQ severity criteria, this exploratory composite was meant to measure cumulative exposure burden rather than clinical severity. Nonetheless, the technique provides a pragmatic descriptive approach that is conceptually compatible with cumulative adversity systems such as the adverse childhood experiences scoring.

The strengths of this study include the use of behavior-specific CSA items instead of a single generic question, which increased the measurement sensitivity and reduced the likelihood of misclassification. Additionally, the study examined patterns and severity of CSA rather than its mere presence or absence, facilitating a more detailed description of exposure. Furthermore, the sample size was sufficiently large to enable subgroup comparisons and to identify statistically significant associations of CSA exposure and various sociodemographic factors. Best-practice guidelines also mention the need for culturally appropriate disclosure procedures in the investigation of CSA in traditional settings. The underreporting of abuse experiences in certain contexts may be influenced by social stigma, fear of judgment, and confidentiality concerns. The use of anonymous online data collection and culturally sensitive survey language in this study may have enhanced disclosure viability while reducing participant discomfort. These variables may be particularly pertinent to future survivor-support strategies, reporting, and screening strategies in Saudi Arabia and other relevant cultural contexts [41,42].

Despite these strengths, the study has several limitations that warrant consideration. The cross-sectional methodology precludes causal inference and does not establish a temporal sequence between CSA exposure and adult sociodemographic outcomes. Additionally, CSA exposure was assessed using retrospective self-reports, which could result in recall bias and underreporting, especially considering the sensitive nature of sexual abuse. Similarly, social desirability and stigma may have contributed to disclosure bias, particularly among men. Moreover, the current approach of severity categorization differs from multidimensional CSA measures that have been standardized worldwide, potentially limiting direct comparison with investigations in different populations and contexts.

Selection bias may have also arisen from the online community-based recruiting method, which focused on individuals who are digitally engaged, have internet access, and are willing to discuss sensitive experiences. Consequently, digitally underprivileged individuals, such as those with limited internet access or lower digital literacy, may have been underrepresented. This underrepresentation could affect sample representativeness and limit the generalizability of the reported prevalence estimates to the broader community of individuals with hypertension in Saudi Arabia. In addition, the absence of a normotensive comparison group and the inclusion of only individuals who had reported hypertension are significant limitations of this study. Consequently, we were unable to determine whether CSA exposure is associated with an increased risk of incident hypertension.

Our findings may have potential clinical and public health implications. The relatively high prevalence of CSA, as well as the occurrence of higher severity exposure in a significant subset, highlights the possible importance of trauma-informed awareness in adult healthcare settings, particularly in settings with chronically ill patients. The findings may help guide future research, clinical considerations, and broad public health measures. The findings also support the potential benefit of trauma-informed clinical interventions in chronic illness care settings.

Best-practice trauma-informed models highlight that healthcare systems should acknowledge the possibility of the long-term impact of childhood trauma on physical and psychological health outcomes. Incorporating trauma-informed concepts into hypertension and primary healthcare services may enhance identification of those with histories of childhood adversity while providing opportunities for more holistic and patient-centered treatment approaches [41,43]. Potential best-practice measures include trauma-sensitive communication training for healthcare providers, confidential screening protocols, multidisciplinary teamwork, and referral mechanisms for psychological and social support services. These measures could enhance the integration of mental and physical healthcare services while enhancing identification and assistance for individuals with histories of childhood trauma [38,41,43].

Future research should employ longitudinal designs to elucidate temporal pathways, incorporate validated trauma instruments, and investigate dose–response relationships between CSA severity and specific health outcomes. Mixed-methods and qualitative approaches may also enhance understanding of disclosure barriers and cultural context.

## 5. Conclusions

A considerable proportion of participants in this study reported experiencing various patterns of CSA at varying levels of severity. Sex, age, marital status, education, and occupation were associated with CSA exposure. Higher cumulative CSA exposure was assigned a greater severity score. These results demonstrate the burden of reported CSA among adults with hypertension and suggest the need for trauma-informed assessment and awareness in healthcare settings serving this population. The findings may help inform future research, clinical consideration, and public health discussions. The cross-sectional design and absence of a reference group in this study hindered conclusions on CSA as a risk factor for hypertension. Thus, future longitudinal research is necessary to ascertain causal pathways and dose–response effects.

## Figures and Tables

**Table 1:** Sociodemographic characteristics of the participants (N = 353).

Variable	*n* (%)
**Sex**	
Male	89 (15.2%)
Female	264 (74.8%)
**Age (year)**	
18–25	110 (31.2%)
26–35	44 (12.5%)
36–49	23 (6.5%)
≥50	176 (49.9%)
**Marital Status**	
Single	132 (37.4%)
Married	155 (43.9%)
Divorced	66 (18.7%)
**Educational Status**	
Secondary school or below	110 (31.2%)
Bachelor’s degree	220 (62.3%)
Higher degree	23 (6.5%)
**Occupational Status**	
Employed	67 (19%)
Unemployed	44 (12.5%)
Retired	132 (37.4%)
Student	110 (31.2%)

**Table 2:** Prevalence, pattern, and severity of childhood sexual abuse (CSA).

CSA Pattern	*n* (%)
**Sexual touching or harassment in childhood**	
Yes	95 (26.9%)
No	258 (73.1%)
**Threatened/coerced into sexual activity (childhood)**	
Yes	61 (17.3%)
No	292 (82.7%)
**Forced sexual acts or exposure to sexual acts (childhood)**	
Yes	66 (18.7%)
No	287 (81.3%)
**Other sexual abuse acts (childhood)**	
Yes	89 (25.2%)
No	264 (74.8%)
**Sexual assault/rape before diagnosis**	
Yes	73 (20.7%)
No	280 (79.3%)
**Any CSA**	
Yes	149 (42.2%)
No	204 (57.8%)
**CSA severity**	
Score: 0	204 (57.8%)
Score: 1	84 (23.8%)
Score: 2	6 (1.7%)
Score: 3	21 (5.9%)
Score: 4	38 (10.8%)

**Table 3:** Associations between childhood sexual abuse and sociodemographic characteristics.

Variable	CSA Absent n (%)	CSA Present n (%)	χ2 *p*-Value
**Sex**			14.92 <0.001
Male	67 (32.8%)	22 (14.8%)	
Female	137 (67.2%)	127 (85.2%)	
**Age (year)**			<0.001
18–25	66 (32.4%)	44 (29.5%)	
26–35	25 (12.3%)	19 (12.8%)	
36–49	23 (11.3%)	0 (0%)	
≥50	90 (44.1%)	86(57.7%)	
**Marital Status**			78.86
Single	110 (53.9%)	22 (14.8%)	
Married	50 (24.5%)	105 (70.5%)	
Divorced	44 (21.6%)	22 (14.8%)	
**Educational Status**			<0.001
Secondary school or below	66 (32.4%)	44 (29.5%)	
Bachelor’s degree	115(56.4%)	105 (70.5%)	
Higher degree	23 (11.3%)	0 (0%)	
**Occupational Status**			<0.001
Employed	45 (22.1%)	22 (14.8%)	
Unemployed	3 (1.5%)	41. (27.5%)	
Retired	68(33.3%)	64 (43%)	
Student	88 (43.1%)	22 (14.8%)	

Among participants reporting any form of childhood sexual abuse.

**Table 4:** Multivariable logistic regression analysis of factors associated with sexual abuse history.

Variable	B	Adjusted OR	95% CI	*p*-Value
Male sex	1.54	4.68	2.45–8.95	<0.001
Married	2.48	11.96	6.56–21.81	<0.001
Employed	0.49	1.64	0.83–3.26	0.157
Constant	−2.77	0.63	-	<0.001

## Data Availability

Anonymized data are accessible upon a reasonable request and subsequent approval by the ethics committee.
